# Association Between CYP2C19 Genotypes With Clinical Phenotypes and Adipokine Levels Among Ischemic Stroke Patients: A Prospective Observational Study

**DOI:** 10.7759/cureus.39265

**Published:** 2023-05-20

**Authors:** Jitender Gairolla, Dheeraj Khurana, Phulen Sarma, Rupinder Kler, Bikash Medhi, Madhu Khullar, Manish Modi, Priyanka Naithani, Ashok Kumar

**Affiliations:** 1 Microbiology, All India Institute of Medical Sciences, Rishikesh, IND; 2 Neurology, Postgraduate Institute of Medical Education and Research, Chandigarh, IND; 3 Pharmacology, All India Institute of Medical Sciences, Guwahati, IND; 4 Genetics, Dayanand Medical College & Hospital, Ludhiana, IND; 5 Pharmacology, Postgraduate Institute of Medical Education and Research, Chandigarh, IND; 6 Experimental Medicine and Biotechnology, Postgraduate Institute of Medical Education and Research, Chandigarh, IND; 7 Nursing, National Institute of Nursing Education, Postgraduate Institute of Medical Education and Research, Chandigarh, IND

**Keywords:** mutation, polymorphism, leptin, ischemic stroke, cyp system, adiponectin, adipocytokines

## Abstract

Background

Cytochrome P450 system is implicated in vascular pathologies, including stroke. Besides its role as a drug metabolizer, it also plays an important role in the metabolism of several endogenous substances like fatty acids, arachidonic acid, etc., which have pro-inflammatory effects. On the other hand, leptin and adiponectin are two of the most common adipose tissue-derived cytokines (adipokines), which are pro-inflammatory and anti-inflammatory in nature, respectively. Both of them are implicated in the pathogenesis of stroke.

Methods

We prospectively recruited ischemic stroke patients (within three months of occurrence of an attack of stroke). The occurrence of composite outcome (recurrence of transient ischemic attack/ischemic stroke or death) was evaluated for association with genetic variants of CYP2C19 (allele *2, *17, *3, and *4, i.e., single nucleotide polymorphism (SNP) 1/2/3/4, identified using TaqMan assays and DNA sequencing). Adiponectin and leptin levels were determined using an enzyme-linked immunosorbent assay. Comparisons were made between stroke vs. control patients and between CYP2C19 intermediate metabolizer (IM)/poor metabolizer (PM) vs. extensive metabolizer (EM)/ultra metabolizer (UM) (PM: *2/*2; IM: *1/*2 vs. EM: *1/*1; UM: *1/*17). P < 0.05 was taken as the threshold for statistical significance.

Results

A total of 204 patients and 101 controls were recruited. With regard to the occurrence of stroke, SNP2 showed a significant positive association. Haplotypes (SNP1/SNP2) AC (OR = 1.75 (1.08-2.83), p = 0.024) and GT (OR = 3.33 (1.53-7.22), p = 0.0026) were strongly associated with the occurrence of ischemic stroke even after adjustment for age and sex (global haplotype association p-value: 0.0062). Haplotype phenotype gender interaction was evident. Among stroke patients, with regard to composite outcome, only SNP1 showed a positive association. The AC haplotype was significantly associated with the occurrence of composite outcome (OR = 2.27 (1.17-4.41), p = 0.016). Among stroke patients, a significant positive association was seen between death and SNP1 (OR = 2.35 (1.13-4.90), p = 0.021) and AC haplotype (OR = 2.73 (1.20-6.22), p = 0.018). However, none of the SNPs or haplotypes showed any association with recurrence.

Significant higher leptin and lower adiponectin levels were observed among stroke patients compared to controls. Leptin levels were higher in IM/PM group. IM/PM phenotypes showed a higher incidence of occurrence of composite outcome (hazard ratio = 2.07 (0.96-4.47), p = 0.056).

Conclusion

CYP2C19 polymorphisms may play a significant role in the pathogenesis of stroke. Leptin could serve as a prominent biomarker of atherosclerosis and inflammation in the early post-stroke period; however, further study is warranted with a larger sample size.

## Introduction

The incidence of stroke is increasing globally. Expected global deaths due to stroke are expected to increase to over 7.8 million by 2030 and produce an immense health burden in the absence of a substantial global public health response [1]. In India, stroke is among the leading causes of disability and death [2]. About 80% of strokes are ischemic in nature [3].

Atherosclerosis and thrombosis are two main processes involved in the pathogenesis of ischemic strokes. Inflammation is one of the major underlying mechanisms for the occurrence of atherosclerosis [3]. Adipocytokines, vasoregulatory molecules, and various growth factors are enormously involved in the intricate process of atherosclerosis [4,5]. Leptin and adiponectin are the most common adipose tissue-derived cytokines that have shown an association with the occurrence of atherosclerosis and stroke, respectively [6].

The cytochrome P450 (CYP) system is a large group of enzymes expressed predominantly in the liver and majorly responsible for drug metabolism. Besides metabolizing, some enzymes of CYP are also involved in the modulation of vascular flow and metabolism of several endogenous substances, such as fatty acids and cholesterol, including arachidonic acid, which has pro-inflammatory effects [7-9]. CYP2C9/19 polymorphism is reported to be associated with atherosclerosis. Also, CYP2C19 has a defined role in cholesterol elimination as well as the elimination of steroid hormones and is being increasingly considered a mediator of inflammation-mediated vascular events. Taking this into consideration, we hypothesized that the common genetic variants of CYP2C19 could affect the dysregulation of adipocytokine levels and may have a possible role in the occurrence and recurrence of stroke in the Indian population.

## Materials and methods

Primary objectives

The primary objectives were as follows: (1) to study the dysregulation of adipocytokines (leptin and total adiponectin) in ischemic stroke patients; (2) to study the association between polymorphisms in CYP2C19 system and occurrence of stroke; (3) to evaluate the association between polymorphisms in CYP2C19 system and occurrence of the composite outcome of recurrence of transient ischemic attack (TIA)/stroke and death.

Secondary outcomes

The secondary objectives were as follows: (1) to evaluate the association between polymorphisms in the CYP2C19 system and the occurrence of the final outcome (live vs. death); (2) to evaluate the association between polymorphisms in the CYP2C19 system and recurrence of TIA/ischemic stroke among live patients; (3) to study the impact of CYP2C19 polymorphisms (allele *2 (rs424485), allele *3 (rs4986893), allele *4 (rs28399504), and allele *17 (rs12248560)) on the production of adipocytokines production; (4) to study the effect of adipocytokines (leptin and total adiponectin) levels on clinical outcome (occurrence of stroke); (5) to study the level of adipocytokines (leptin and total adiponectin) among poor metabolizer (PM)/intermediate metabolizer (IM) and extensive metabolizer (EM)/ultra metabolizer (UM).

Definition of composite clinical outcome

In our study, the composite outcome was defined as the composite of the occurrence of any of the following events: death, recurrent TIAs, or ischemic events.

Subject selection

A prospective study was carried out on ischemic stroke/TIA patients who presented within three months of an attack. The patients were recruited from indoor/outdoor patient services of the Postgraduate Institute of Medical Education and Research, Chandigarh, India.

Study population

Regarding the inclusion of cases, we included patients ≥ 18 years of age of either gender presenting with TIA or ischemic stroke occurring within the last three months (of either anterior or posterior circulation, including lacunars strokes, confirmed by an imaging modality, CT scan, or MRI) and who were on secondary prophylaxis for stroke with either clopidogrel or clopidogrel and aspirin. We excluded patients with intracranial hemorrhagic stroke, cardioembolic stroke, and non-atherosclerotic stroke, e.g., dissection and vasculitis. Patients with chronic disease, cancer, collagen vascular disease, HIV, congestive cardiac failure, pregnancy, and contrast allergy were also excluded.

Control group

Age and sex-matched unrelated controls without any history of stroke and who were not taking any anti-inflammatory drug at least for 14 days were enrolled.

Follow up

The TOAST (Trial of ORG 10172 in Acute Stroke Treatment) criteria [10] were used to classify the stroke subtypes. They were followed up telephonically and patients were queried for any recurrence of ischemic stroke, TIAs, and death due to vascular causes. The median follow-up time was 34 months (IQR: 19-46).

Clinical data collection

All data collected were assessed by two neurologists separately in a blinded manner. The standard criteria followed for the assessment of hypertension, diabetes mellitus, smoking, and obesity have been mentioned in a previous report by Gairolla et al. [11].

Sample collection and genotyping

Five milliliters of whole blood was collected from each patient in ethylenediaminetetraacetic acid (EDTA) and sodium (Na)-heparin vials. DNA extraction was performed in an EDTA sample using PureLink Genomic DNA Kit (Invitrogen, Carlsbad, CA). Genetic variants of CYP2C19 (allele *2 (rs424485), allele *3 (rs4986893), allele *4 (rs28399504), and allele *17 (rs12248560)) were genotyped using TaqMan-based single nucleotide polymorphism (SNP) genotyping assays on real-time polymerase chain reaction (PCR) machine (Applied Biosystems StepOnePlus, Thermo Fisher Scientific, Waltham, MA) as per manufacturer guidelines (Supplementary Table A1). Five microliters of TaqMan universal PCR master and DNA range from 5 to 25 ng were added to the final volume of 10 μl (Supplementary Table A2). PCR data were analyzed using StepOne software version 2.2.2. DNA sequencing for CYP2C19*3 (ABI 3730xl DNA Analyzer, SciGenom Labs, Kochi, India) was performed in randomly selected samples.

Adipocytokine measurement (leptin and total adiponectin)

Estimation of adipocytokines levels (leptin (Cat. # DLP00) and total adiponectin (Cat. # DRP 300)) was carried out in plasma sample pull out from sodium (Na)-heparin blood using quantitative enzyme-linked immunosorbent immunoassay technique purchased from R&D Systems (Minneapolis, MN). Plasma levels of leptin and total adiponectin (n = 204) were compared between patients (n = 204) and healthy controls (n = 50). A linearity curve was prepared for the calculation of plasma concentration using a linear equation model. Sensitivity for leptin and total adiponectin were less than 7.8 pg/ml and 0.246 ng/ml, respectively. Intra-assay variation (coefficient of variation) for leptin (1 ng/ml) and total adiponectin (125 ng/ml) was 8.6% and 14.1%, respectively.

Ethical permission

Informed consent was signed by all recruited patients after ethical approval was obtained from the Institutional Ethical Committee (IEC), Postgraduate Institute of Medical Education and Research. The IEC approved the study (NK/1216//Ph.D/20259, INT/IEC/2015/706).

Sample size

The sample size was calculated on the basis of the genetic frequency of CYP2C19 allele *2 in the Indian population keeping 29% as frequency and confidence limits at 7%. A minimum sample size of 162 (95% CI) patients is required to accomplish the study objectives. While calculating the sample size, we followed the guideline of the Department of Biotechnology, India. We recruited 101 healthy controls and 204 stroke patients for the study.

Statistical analysis

Categorical variables are expressed as percentages (%) and continuous variables, which are not normally distributed, are described in the median with interquartile range (IQR), and a non-parametric test (Mann-Whitney test) was done to determine the significance. Any deviation of allelic and genotype distribution from Hardy-Weinberg equilibrium (HWE) proportions was tested using χ2 or Fisher's exact test. Cox regression and survival analysis with Kaplan-Meier curves were applied to calculate the hazard ratio and cumulative event-free survival. Multivariable Cox regression analysis was done in variables that were statistically significant in univariate analysis. For base numbering and allele definitions of CYP2C, we followed the nomenclature of the Human Cytochrome P450 (CYP) Allele Nomenclature Committee (www.cypalleles.ki.se) [12]. The genotype-phenotype associations were evaluated using both univariate and multivariate approaches using different models for genetic association studies (co-dominant, dominant, recessive, over-dominant, and log additive models). In the case of two SNPs or more, linkage disequilibrium and haplotype association were evaluated with the clinical outcomes.

Poor/intermediate metabolizers and extensive/ultra metabolizers with CYP2C19 function description

Based on CYP2C19 genotypes (allele *2 (rs424485), allele *3 (rs4986893), allele *4 (rs28399504), and allele *17 (rs12248560)), patients were categorized into two groups as poor/intermediate metabolizers (PM: *2/*2; IM: *1/*2) and extensive/ultra metabolizers (EM: *1/*1; UM: *1/*17) [12]. Heterozygotes with one mutated allele and other wild types of allele (*1/*2) are intermediate metabolizers and homozygous mutated states (*2/*2)/heterozygous mutated (e.g., *2/*3) are poor metabolizers [12].

The wild-type CYP2C19*1 allele is associated with functional CYP2C19-mediated metabolism. The most common CYP2C19 loss-of-function (LOF) allele is *2 (c.681G>A; rs4244285). Other CYP2C19 variant alleles with reduced or absent enzymatic activity have been identified (e.g., *3-*8) [12]. The CYP2C19 LOF alleles are inherited as an autosomal co-dominant trait. The lowest activity is seen in LOF allele homozygotes (*3/*3) or compound heterozygotes (*3/*3), followed by heterozygotes (*1/*2 or *1/*3) compared to normal wild type (*1/*1). In this regard, CY2C19 genotypes are categorized as extensive metabolizers (EMs: *1/*1), intermediate metabolizers (e.g., IMs *1/*2 and *1/*3), or poor metabolizers (PMs: *2/*2 and *2/*3). By contrast, the common CYP2C19*17 allele (c.-806C>T; rs12248560) results in increased activity as a consequence of enhanced transcription. However, *2/*17 compound heterozygotes should be classified as IMs (significant linkage disequilibrium exists between *2 and *17 alleles) [12].

## Results

Demographic and clinical characteristics of the patients

**Figure 1:**
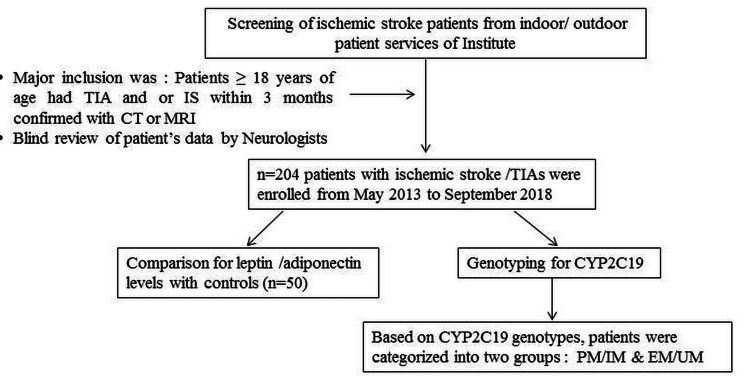
Study flowchart TIA: transient ischemic attack; IS: ischemic attack; PM: poor metabolizer; IM: intermediate metabolizer; EM: extensive metabolizer; UM: ultra metabolizer.

A total of 204 patients with ischemic stroke/TIAs were enrolled from May 2013 to September 2018. The mean age (SD) of stroke patients was 57 ± 13 years and 156 (76.5%) patients were male. Of the patients, 184 (90.2%) had an ischemic stroke. A total of 133 (65.2%) patients had a history of hypertension and 60 (29.4%) were smokers. Of the patients, 66 (32.4%) had diabetes mellitus and 28 (13.7%) had obesity. A family history of stroke was present in 12.7% of patients (Table 1). All the patients belonged to North India and the majority were primarily from Chandigarh (union territory) (41.1%) and the state of Punjab (27.5%). Figure 1 depicts the study flowchart.

**Table 1:** Demographic characteristics * Definition used Hypertension: Patients are considered hypertensive if they are taking antihypertensive agents or have a systolic blood pressure of 140 mm Hg or higher and a diastolic blood pressure of 90 mm Hg or higher. Diabetes mellitus: Patients on oral hypoglycemic agents (OHAs) or insulin or having a fasting plasma glucose level above 126 mg/dl are considered as having diabetes mellitus. Current smoker: Patients who are current smokers or who had quit smoking for less than three years are considered smokers. BMI: Overweight and obesity were determined according to the WHO guidelines. IS: ischemic attack; TIA: transient ischemic attack; DM: diabetes mellitus; CAD: coronary artery disease.

Characteristics		n = 204
Age (years, mean ± SD)		57 ± 13
Gender (n, %)	Male	156 (76.5%)
	Female	48 (23.5%)
Type of stroke	Ischemic stroke (n, %)	184 (90.2%)
	TIAs (n, %)	20 (9.8%)
Risk factors		
Hypertension (n, %)		133 (65.2%)
Recurrent IS (n, %)		45 (22.1%)
Recurrent TIAs (n, %)		16 (7.8%)
Diabetes mellitus (n, %)		66 (32.4%)
Current smokers (n, %)		60 (29.4%)
Alcohol intake (n, %)		43 (21.1%)
BMI (kg/m^2^)		24 (21.5-27.1)
25-30 (kg/m^2^; n, %)		44 (21.6%)
>30 (kg/m^2^; n, %)		28 (13.7%)
Family history of DM (n, %)		45 (22.1%)
Family history of stroke (n, %)		26 (12.7%)
Family history of hypertension (n, %)		36 (17.6%)
CAD (n, %)		38 (18.6%)

Genotype frequencies

Minor allele frequency (MAF) (%) of CYP2C19*2 (G>A; rs424485, SNP1) and CYP2C19*17 (C>T; rs12248560, SNP2) was 34.5% and 21.5%, respectively. Mutant and heterozygous variants of CYP2C19*3 (G>A; rs4986893, SNP3) and *4 (G>A; rs28399504, SNP4) alleles were not reported. All reported alleles and genotypes were in HWE (p > 0.05). No compound heterozygous of CYP2C19 (*2/*17) was reported. The observed genotype frequencies are mentioned in Table 2. The linkage disequilibrium plot is evaluated between SNP1 and SNP2. However, no significant linkage disequilibrium was seen among the two SNPs. Data are presented in Table 3.

**Table 2:** Observed genotype frequencies of CYP2C19 system 0* indicates the absence of hetero or mutant forms of genotype. SNP: single nucleotide polymorphism.

Polymorphisms	Genotype	Allele nomenclature	Number and percentage, n (%)
CYP2C19*2, (G>A); rs424485 SNP1	GG	*1/*1	85 (41.7%)
	GA	*1/*2	97 (47.5%)
	AA	*2/*2	22 (10.8%)
CYP2C19*17, (C>T); rs12248560 SNP2	CC	*1/*1	123 (60.3%)
	CT	*1/*17	74 (36.3%)
	TT	*17/*17	7 (3.4%)
CYP2C19*3, (G>A); rs4986893 SNP3	GG	*1/*1	204 (100%)
	GA	*1/*3	0*
	AA	*3/*3	0*
CYP2C19*4, (G>A); rs28399504 SNP4	GG	*1/*1	204 (100%)
	GA	*1/*4	0*
	AA	*4/*4	0*

**Table 3:** Pairwise comparison of measures of D, D′, r, and p-values for CYP2C19 polymorphisms in the present study SNP: single nucleotide polymorphism.

	SNP1 CYP2C19*2	SNP-2 CYP2C19*17
SNP1 CYP2C19*2	-	-0.0303, 0.468, -0.1628 1e-04
SNP2 CYP2C19*17	-	-

Impact of SNP on the occurrence of stroke: stroke vs. no stroke (control)

Stroke vs. No Stroke (Control): Allelic Frequency, Genotype Frequency, and Hardy-Weinberg Equilibrium

The details of allelic and genotype frequencies of HWE p-values of SNP1 and SNP2 are shown in Table 4.

**Table 4:** Stroke vs. no stroke (control): allelic frequency, genotype frequency, and Hardy-Weinberg equilibrium SNP: single nucleotide polymorphism; HWE: Hardy-Weinberg equilibrium.

SNP	Allele/genotype		All subject		Control		Stroke	
			Count	Proportion	Count	Proportion	Count	Proportion
SNP1	Allele	G	404	0.66	137	0.68	267	0.65
		A	206	0.34	65	0.32	141	0.35
	Genotype	A/A	30	0.1	8	0.08	22	0.11
		G/A	146	0.48	49	0.49	97	0.48
		G/G	129	0.42	44	0.44	85	0.42
	HWE		0.25		0.36		0.54	
SNP2	Allele	C	493	0.81	173	0.86	320	0.78
		T	117	0.19	29	0.14	88	0.22
	Genotype	C/C	195	0.64	72	0.71	123	0.60
		C/T	103	0.34	29	0.29	74	0.36
		T/T	7	0.02	0	0	7	0.03
	HWE		0.14		0.21		0.41	

Stroke vs. No Stroke (Control): Genetic Association

Regarding CYP genotypes as predictors for the occurrence of stroke, we used different genetic models for the association studies. The models were decided on the basis of the Akaike information criterion (AIC) and Bayes information criterion (BIC) values of that respective model. No significant association was observed between the occurrence of stroke and SNP1.

In the case of SNP2, the lowest AIC value was 385.9 and the lowest BIC value was 401.2. The models performing within two units of this lowest AIC and BIC value were recessive and the log additive model. Interestingly, SNP2 showed a significant positive association with the occurrence of stroke in both of these two models. Data are presented in Table 5.

**Table 5:** Stroke vs. no stroke (control): genetic association data (n = 305, adjusted by sex and age) SNP: single nucleotide polymorphism; AIC: Akaike information criterion; BIC: Bayes information criterion.

SNP	Model	Genotype	Control	Stroke	OR	P-value	AIC	BIC
SNP1	Co-dominant	G/G	44 (43.6%)	85 (41.7%)	1.00	0.55	393	411.6
		A/G	49 (48.5%)	97 (47.5%)	1.00 (0.61-1.66)			
		A/A	8 (7.9%)	22 (10.8%)	1.60 (0.65-3.93)			
	Dominant	GG	44 (43.6%)	85 (41.7%)	1.00	0.75	392.1	407
		A/G-A/A	57 (56.4%)	119 (58.3%)	1.08 (0.67-1.76)			
	Recessive	G/G-A/G	93 (92.1%)	182 (89.2%)	1.00	0.28	391	405.9
		A/A	8 (7.9%)	22 (10.8%)	1.60 (0.67-3.78)			
	Over dominant	G/G-A/A	52 (51.5%)	107 (52.5%)	1.00	0.75	392.1	407
		A/G	49 (48.5%)	97 (47.5%)	0.92 (0.57-1.50)			
	Log additive	--	--	--	1.15 (0.79-1.67)	0.46	391.7	406.5
SNP2	Co-dominant	C/C	72 (73.1%)	123 (60.3%)	1.00	0.016	385.9	404.5
		C/T	29 (28.7%)	74 (36.3%)	1.51 (0.89-2.55)			
		T/T	0 (0%)	7 (3.4%)	NA (0.00-NA)			
	Dominant	C/C	72 (71.3%)	123 (60.3%)	1.00	0.053	388.5	403.4
		C/T-T/T	29 (28.7%)	81 (39.7%)	1.66 (0.99-2.79)			
	Recessive	C/C-C/T	101 (100%)	197 (96.6%)	1.00	0.015	386.3	401.2
		T/T	0 (0%)	7 (3.4%)	NA (0.00-NA)			
	Over dominant	C/C-T/T	72 (71.3%)	130 (63.7%)	1.00	0.18	390.4	405.3
		C/T	29 (28.7%)	74 (36.3%)	1.43 (0.84-2.41)			
	Log additive				1.75 (1.07-2.84)	0.021	386.9	401.7

Occurrence of Stroke: Sex and SNP Covariate Interaction

To evaluate the impact of age and sex, we further evaluated the association in the light of multivariate analysis. When adjusted by age, no significant interaction was seen between SNP and sex with the occurrence of stroke. Data are presented in Tables 6, 7.

**Table 6:** Occurrence of stroke: sex and SNP covariate interaction (sex within SNP) SNP: single nucleotide polymorphism.

	Sex within SNP (n = 305, adjusted by age)				
SNP1			Control	Stroke	OR
	G/G	Female	11	21	1.00
		Male	33	64	1.02 (0.44-2.38)
	A/G	Female	12	21	1.00
		Male	37	76	1.15 (0.51-2.60)
	A/A	Female	5	6	1.00
		Male	3	16	4.53 (0.81-25.25)
	Test for interaction in the trend = 0.27				
SNP2	C/C	Female	24	31	1.00
		Male	48	92	1.45 (0.77-2.76)
	C/T	Female	4	14	1.00
		Male	25	60	0.69 (0.21-2.30)
	T/T	Female	0	3	1.00
		Male	0	4	1.00
	Test for interaction in the trend = 0.2				

**Table 7:** Occurrence of stroke: sex and SNP covariate interaction (SNP within sex) SNP: single nucleotide polymorphism.

SNP	Gender	Genotype	Control	Stroke	OR
SNP1	Female	G/G	11	21	1.00
		A/G	12	21	0.92 (0.33-2.55)
		A/A	5	6	0.68 (0.17-2.77)
	Male	G/G	33	64	1.00
		A/G	37	76	1.03 (0.58-1.85)
		A/A	3	16	3.03 (0.81-11.26)
	Test for interaction in the trend = 0.28				
SNP2	Female	C/C	24	31	1.00
		C/T	4	14	2.74 (0.80-9.45)
		T/T	0	3	--
	Male	C/C	48	92	1.00
		C/T	25	60	1.30 (0.72-2.33)
		T/T	0	4	--
	Test for interaction in the trend = 0.55				

Haplotype Association With Stroke

The AC haplotype (OR = 1.75 (1.08-2.83), p = 0.024) and GT haplotype (OR = 3.33 (1.53-7.22), p = 0.0026) were found to be strongly associated with the occurrence of stroke (global haplotype association p-value: 0.0062). Data are presented in Table 8.

**Table 8:** Haplotype association with stroke SNP: single nucleotide polymorphism.

S. no	SNP1	SNP2	Frequency	OR	P-value
1	G	C	0.5108	1.00	--
2	A	C	0.2974	1.75 (1.08-2.83)	0.024
3	G	T	0.1515	3.33 (1.53-7.22)	0.0026
4	A	T	0.0403	0.64 (0.22-1.88)	0.42
Global haplotype association p-value: 0.0062					

Among females, the haplotype phenotype interaction was not observed; however, haplotype phenotype interaction was evident among males (Table 9), and compared to the GC haplotype, AC and GT haplotypes were significantly associated with the occurrence of stroke (after adjusting for age).

**Table 9:** Haplotype and interaction with sex (adjusted by age)

		Haplotype within sex		Sex within haplotype	
Haplotype	Frequency	Female	Male	Female	Male
		OR (95% CI)	OR (95% CI)	OR (95% CI)	OR (95% CI)
GC	0.513	1.00	1.00	1.00	1.02 (0.40-2.61)
AC	0.2952	1.32 (0.62-2.83)	2.15 (1.17-3.94)	1.00	1.67 (0.82-3.39)
AT	0.0425	0.59 (0.10-3.50)	0.60 (0.17-2.13)	1.00	1.03 (0.12-8.93)
GT	0.1493	Inf.	2.99 (1.28-7.00)	1.00	0.00 (-inf-inf)

Stroke patients: composite outcome

Allelic Frequency, Genotype Frequency, and HWE Among Stroke Patients With Respect to the Occurrence of Composite Outcome

The details of allelic and genotype frequencies of HWE p-values of SNP1 to SNP4 with reference to the occurrence of composite outcomes among stroke patients are shown in Table 10.

**Table 10:** Allelic frequency, genotype frequency, and HWE among stroke patients with respect to the occurrence of the composite outcomes SNP: single nucleotide polymorphism; HWE: Hardy-Weinberg equilibrium.

SNP	Allele/genotype		All subjects (n = 204)		No composite outcome		Composite outcome present	
			Count	Proportion	Count	Proportion	Count	Proportion
SNP1	Allele	G	267	0.65	230	0.67	37	0.56
		A	141	0.35	112	0.33	29	0.44
	Genotype	A/A	22	0.11	17	0.1	5	0.15
		G/A	97	0.48	78	0.46	19	0.58
		G/G	85	0.42	76	0.44	9	0.27
	HWE		0.54		0.73		0.48	
SNP2	Allele	C	320	0.78	265	0.77	55	0.83
		T	88	0.22	77	0.23	11	0.17
	Genotype	C/C	123	0.6	100	0.58	23	0.7
		C/T	74	0.36	65	0.38	9	0.27
		T/T	7	0.03	6	0.04	1	0.03
	HWE		0.41		0.38		1	

All Stroke Patients: Genetic Association Data (n = 305, Adjusted by Sex and Age)

SNP1: Among stroke patients, while evaluating the association between SNP and occurrence of the composite outcome, the best-performing models in terms of lowest AIC and BIC score were the dominant model (2.31 (0.99-5.35), p = 0.043) and log-additive model (1.82 (1.02-3.27), p = 0.042). Interestingly, both these models showed a positive association between SNP1 and the occurrence of composite outcomes. Data are presented in Table 11.

**Table 11:** Stroke patients: genetic association data (n = 305, adjusted by sex and age) SNP: single nucleotide polymorphism; AIC: Akaike information criterion; BIC: Bayes information criterion.

SNP	Model	Genotype	No composite	Composite	OR	P-value	AIC	BIC
SNP1	Co-dominant	G/G	76 (44.4%)	9 (27.3%)	1.00	0.11	179.7	196.3
		A/G	78 (45.6%)	19 (57.6%)	2.17 (0.91-5.19)			
		A/A	17 (9.9%)	5 (15.2%)	3.03 (0.86-10.65)			
	Dominant	GG	76 (44.4%)	9 (27.3%)	1.00	0.043	178	191.3
		A/G-A/A	95 (55.6%)	24 (72.7%)	2.31 (0.99-5.35)			
	Recessive	G/G-A/G	154 (90.1%)	28 (84.8%)	1.00	0.27	180.9	194.2
		A/A	17 (9.9%)	5 (15.2%)	1.91 (0.63-5.81)			
	Over dominant	G/G-A/A	93 (54.4%)	14 (42.4%)	1.00	0.20	180.5	193.8
		A/G	78 (45.6%)	19 (57.6%)	1.64 (0.76-3.53)			
	Log additive	--	--	--	1.82 (1.02-3.27)	0.042	178	191.3
SNP2	Co-dominant	C/C	100 (58.5%)	23 (69.7%)	1.00	0.61	183.1	199.7
		C/T	65 (38%)	9 (27.3%)	0.66 (0.28-1.54)			
		T/T	6 (3.5%)	1 (3%)	0.71 (0.08-6.37)			
	Dominant	C/C	100 (58.5%)	23 (69.7%)	1.00	0.32	181.1	194.4
		C/T-T/T	71 (41.5%)	10 (30.3%)	0.67 (0.29-1.51)			
	Recessive	C/C-C/T	165 (96.5%)	32 (97%)	1.00	0.85	182.1	195.4
		T/T	6 (3.5%)	1 (3%)	0.81 (0.09-7.18)			
	Over dominant	C/C-T/T	106 (62%)	24 (72.7%)	1.00	0.35	181.2	194.5
		C/T	65 (38%)	9 (27.3%)	0.67 (0.29-1.56)			
	Log additive				0.72 (0.35-1.47)	0.35	181.3	194.5

SNP2: The best-performing model in terms of AIC and BIC is the dominant model. However, no association was seen between SNP2 and the occurrence of composite outcomes. Data are presented in Table 11.

Occurrence of Composite Outcome: Sex and SNP Covariate Interaction

In terms of the occurrence of composite outcome and the impact of the important covariate (sex), no significant impact of sex was observed on the occurrence of the composite outcome. Data are presented in Tables 12, 13.

**Table 12:** Occurrence of composite outcome: sex and SNP covariate interaction (sex within SNP) SNP: single nucleotide polymorphism.

	Sex within SNP (n = 305, adjusted by age)				
SNP1			No composite	Composite	OR
	G/G	Female	18	3	1.00
		Male	58	6	0.68 (0.15-3.06)
	A/G	Female	15	6	1.00
		Male	63	13	0.51 (0.16-1.60)
	A/A	Female	4	2	1.00
		Male	13	3	0.50 (0.06-4.23)
	Test for interaction in the trend = 0.79				
SNP2	C/C	Female	22	9	1.00
		Male	78	14	0.46 (0.17-1.22)
	C/T	Female	13	1	1.00
		Male	52	8	2.04 (0.23-18.10)
	T/T	Female	2	1	1.00
		Male	4	0	0.00
	Test for interaction in the trend = 0.76				

**Table 13:** Occurrence of composite outcome: sex and SNP covariate interaction (SNP within sex) SNP: single nucleotide polymorphism.

SNP	Gender	Genotype	No composite	Composite	OR
SNP1	Female	G/G	18	3	1.00
		A/G	15	6	2.64 (0.54-12.78)
		A/A	4	3	3.72 (0.44-31.36)
	Male	G/G	58	6	1.00
		A/G	63	13	1.98 (0.70-5.62)
		A/A	13	3	2.74 (0.58-12.81)
	Test for interaction in the trend = 0.95				
SNP2	Female	C/C	22	9	1.00
		C/T	133	1	0.20 (0.02-1.82)
		T/T	2	1	1.39 (0.11-17.53)
	Male	C/C	78	14	1.00
		C/T	52	8	0.90 (0.35-2.32)
		T/T	4	0	0.00
	Test for interaction in the trend = 0.2				

Haplotype Association With the Composite Outcome

While evaluating the different haplotypes of SNP1 and SNP2 and their association with the occurrence of composite outcomes, the AC haplotype was significantly associated with the occurrence of the primary outcome (OR = 2.27 (1.17-4.41), p = 0.016, global haplotype association p-value: 0.044). Data are presented in Table 14.

**Table 14:** Haplotype association with the composite outcome SNP: single nucleotide polymorphism.

S. no.	SNP1	SNP2	Frequency	OR	P-value
1	G	C	0.4669	1.00	--
2	A	C	0.3174	2.27 (1.17-4.41)	0.016
3	G	T	0.1875	1.18 (0.53-2.63)	0.69
4	A	T	0.0282	0.00 (-Inf-Inf)	1
Global haplotype association p-value: 0.044					

While evaluating the interaction between haplotypes, composite outcome, and impact of the important covariate (sex), no significant covariate effect was observed after adjusting for age. Data are presented in Table 15.

**Table 15:** Composite outcome: haplotype and interaction with sex (adjusted by age)

		Haplotype within sex (n = 204)		Sex within haplotype (n = 204)	
Haplotype	Frequency	Female	Male	Female	Male
		OR (95% CI)	OR (95% CI)	OR (95% CI)	OR (95% CI)
GC	0.467	1.00	1.00	1.00	0.69 (0.13-3.79)
AC	0.3173	2.90 (0.85-9.90)	2.05 (0.94-4.48)	1.00	0.49 (0.18-1.33)
AT	0.0282	0.00 (-Inf-Inf)	0.00 (-Inf-Inf)	1.00	Inf
GT	0.1874	1.16 (0.28-4.82)	1.20 (0.46-3.18)	1.00	0.72 (0.16-3.29)

All stroke patients: dead vs. alive

All Stroke Patients: Alive vs. Dead: Allelic Frequency, Genotype Frequency, and HWE

The details of allelic and genotype frequencies of HWE p-values of SNP1 to SNP2 with reference to the occurrence of the final outcome (alive vs. death) among stroke patients are shown in Table 16.

**Table 16:** All stroke patients: alive vs. dead: allelic frequency, genotype frequency, and Hardy-Weinberg equilibrium SNP: single nucleotide polymorphism; HWE: Hardy-Weinberg equilibrium.

SNP	Allele/genotype		All subjects (n = 204)		Alive		Dead	
			Count	Proportion	Count	Proportion	Count	Proportion
SNP1	Allele	G	267	0.65	248	0.67	19	0.5
		A	141	0.35	122	0.33	19	0.5
	Genotype	A/A	22	0.11	17	0.09	5	0.26
		G/A	97	0.48	88	0.48	9	0.47
		G/G	85	0.42	80	0.43	5	0.26
	HWE		0.54		0.4		1.00	
SNP2	Allele	C	320	0.78	287	0.78	33	0.87
		T	88	0.22	83	0.22	5	0.13
	Genotype	C/C	123	0.6	109	0.59	14	0.74
		C/T	74	0.36	69	0.37	5	0.26
		T/T	7	0.03	7	0.04	0	0
	HWE		0.41		0.4		1.00	

All Stroke Patients: Alive vs. Dead: Genetic Association Data

We evaluated the impact of different SNPs and the occurrence of final outcomes (alive vs. dead) among stroke patients using different models of genetic analysis.

SNP1: On the basis of AIC and BIC values, the best model was the log-additive model. A significant positive association was seen between the occurrence of the final outcome (death) and SNP1 (OR = 2.35 (1.13-4.90), p = 0.021). Data are presented in Table 17.

**Table 17:** All stroke patients: alive vs. dead: genetic association data (n = 204, adjusted by sex and age) SNP: single nucleotide polymorphism; AIC: Akaike information criterion; BIC: Bayes information criterion.

SNP	Model	Genotype	Alive	Dead	OR	P-value	AIC	BIC
SNP1	Co-dominant	G/G	80 (43.2%)	5 (26.3%)	1.00	0.054	127.9	144.4
		A/G	88 (47.6%)	9 (47.4%)	1.69 (0.54-5.30)			
		A/A	17 (9.2%)	5 (26.3%)	5.79 (1.44-23.31)			
	Dominant	GG	80 (43.2%)	5 (26.3%)	1.00	0.12	129.3	142.5
		A/G-A/A	105 (56.8%)	14 (73.7%)	2.25 (0.77-6.57)			
	Recessive	G/G-A/G	168 (90.8%)	14 (73.7%)	1.00	0.025	126.7	140
		A/A	17 (9.2%)	5 (26.3%)	4.26 (1.30-13.93)			
	Over dominant	G/G-A/A	97 (52.4%)	10 (52.6%)	1.00	0.97	131.7	144.9
		A/G	88 (47.6%)	9 (47.4%)	0.98 (0.38-2.56)			
	Log additive	--			2.35 (1.13-4.90)	0.021	126.4	139.6
SNP2	Co-dominant	C/C	109 (58.9%)	14 (73.7%)	1.00	0.32	131.4	148
		C/T	69 (37.3%)	5 (26.3%)	0.61 (0.21-1.79)			
		T/T	7 (3.8%)	0 (0%)	0.00 (0.00-NA)			
	Dominant	C/C	109 (58.9%)	14 (73.7%)	1.00	0.26	130.4	143.7
		C/T-T/T	76 (41.1%)	5 (26.3%)	0.55 (0.19-1.60)			
	Recessive	C/C-C/T	178 (96.2%)	19 (100%)	1.00	0.23	130.2	143.5
		T/T	7 (3.8%)	0 (0%)	0.00 (0.00-NA)			
	Over dominant	C/C-T/T	116 (62.7%)	14 (73.7%)	1.00	0.42	131	144.3
		C/T	69 (37.3%)	5 (226.3%)	0.65 (0.22-1.90)			
	Log additive				0.53 (0.20-1.45)	0.19	130	143.2

SNP2: On the basis of AIC and BIC values, the best model was the log-additive model. No association was seen between the occurrence of the final outcome (death) and SNP2 (OR = 0.53 (0.20-1.45), p = 0.19). Data are presented in Table 17.

All Stroke Patients: Alive vs. Dead: Sex and SNP Covariate Interaction

While evaluating for any covariate effect for the important covariate (sex), no significant association was seen after adjustment for sex. Data are presented in Tables 18, 19.

**Table 18:** All stroke patients: alive vs. dead: sex and SNP covariate interaction (sex within SNP) SNP: single nucleotide polymorphism.

	Sex within SNP (n = 204, adjusted by age)				
SNP1			Alive	Dead	OR
	G/G	Female	20	1	1.00
		Male	60	4	1.49 (0.15-14.37)
	A/G	Female	18	3	1.00
		Male	70	6	0.52 (0.12-2.30)
	A/A	Female	4	2	1.00
		Male	13	3	0.50 (0.06-4.22)
	Test for interaction in the trend = 0.45				
SNP2	C/C	Female	26	5	1.00
		Male	83	9	0.59 (0.18-1.94)
	C/T	Female	13	1	1.00
		Male	56	4	0.93 (0.10-9.15)
	T/T	Female	3	0	1.00
		Male	4	0	0.99
	Test for interaction in the trend = 0.65				

**Table 19:** All stroke patients: alive vs. dead: sex and SNP covariate interaction SNP within sex SNP: single nucleotide polymorphism.

SNP	Gender	Genotype	Alive	Dead	OR
SNP1	Female	G/G	20	1	1.00
		A/G	18	3	3.65 (0.34-39.23)
		A/A	4	2	12.71 (0.87-184.88)
	Male	G/G	60	4	1.00
		A/G	70	6	1.26 (0.34-4.73)
		A/A	13	3	4.27 (0.82-22.24)
	Test for interaction in the trend = 0.7				
SNP2	Female	C/C	26	5	1.00
		C/T	13	1	0.43 (0.05-4.15)
		T/T	3	0	0.00
	Male	C/C	83	9	1.00
		C/T	56	4	0.68 (0.20-2.34)
		T/T	4	0	0.00
	Test for interaction in the trend = 0.94				

Haplotype Association With Final Outcome (Alive vs. Dead)

Among different haplotypes, the AC haplotype was found to be associated with death (OR = 2.73 (1.20-6.22), p = 0.018, global haplotype association p-value: 0.039). Data are presented in Table 20.

**Table 20:** Haplotype association with final outcome (alive vs. dead) SNP: single nucleotide polymorphism.

S. no.	SNP1	SNP2	Frequency	OR	P-value
1	G	C	0.4673	1.00	--
2	A	C	0.3170	2.73 (1.20-6.22)	0.018
3	G	T	0.1871	0.96 (0.32-2.88)	0.94
4	A	T	0.0286	0.00 (-Inf-Inf)	1
Global haplotype association p-value: 0.039					

No significant interaction was seen between the occurrence of the final outcome, haplotype, and sex (after adjustment by age). Data are shown in Supplementary Table A3.

All alive stroke patients: recurrence vs. no recurrence

Allelic Frequency, Genotype Frequency, and HWE: All Alive Patients: No Recurrence vs. Recurrence

The details of allelic and genotype frequencies of HWE p-values of SNP1 to SNP2 with reference to the recurrence of stroke are shown in Supplementary Table A4.

All Alive Patients: No Recurrence vs. Recurrence: Genetic Association Data

We evaluated the impact of different SNPs and the occurrence of final outcomes (alive vs. dead) among stroke patients using different models of genetic analysis.

SNP1: On the basis of AIC and BIC values, the best model was the recessive model. However, no association was seen with recurrence (OR = 0.38 (0.10-1.40), 0.12). Data are shown in Supplementary Table A5.

SNP2: On the basis of AIC and BIC values, the best model was the over-dominant model. No association was seen between recurrence and SNP2 (OR = 0.83 (0.44-1.59), p = 0.58). Data are shown in Supplementary Table A5.

No significant interaction was seen in the case of both SNP1 and SNP2 and the important covariate (sex) after adjustment of age. Data are shown in Supplementary Tables A6 and A7.

All Alive Stroke Patients: No Recurrence vs. Recurrence: Haplotype Association

Among alive patients, no significant association was seen between any of the haplotypes and recurrence. Data are shown in Supplementary Table A8. No significant covariate effect of sex was observed after adjustment for age. Data are shown in Supplementary Table A9.

Adipokine and leptin levels and their association with stroke

Adipocytokine Levels Between Stroke Patients and Controls

**Figure 2:**
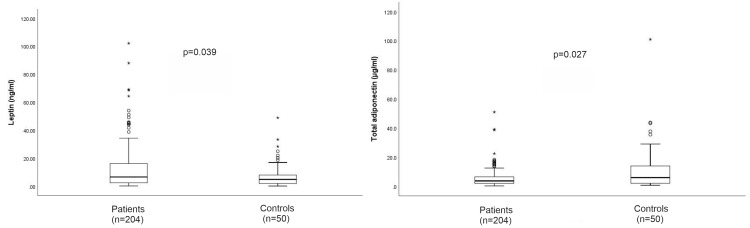
Plasma leptin (ng/ml) and total adiponectin (µg/ml) between patients (n = 204) and controls (n = 50)

Significant differences in leptin (ng/ml) (median (IQR) = 6.6 (2.4-16.2) vs. 4.8 (1.8-8.5), p = 0.039) and total adiponectin levels (µg/ml) (median IQR = 3.6 (1.9-6.4) vs. 5.9 (1.9-14.5), p = 0.027) were observed between patients and controls (Figure 2).

Adiponectin and Leptin Levels Among IM/PM and EM/UM Groups

**Figure 3:**
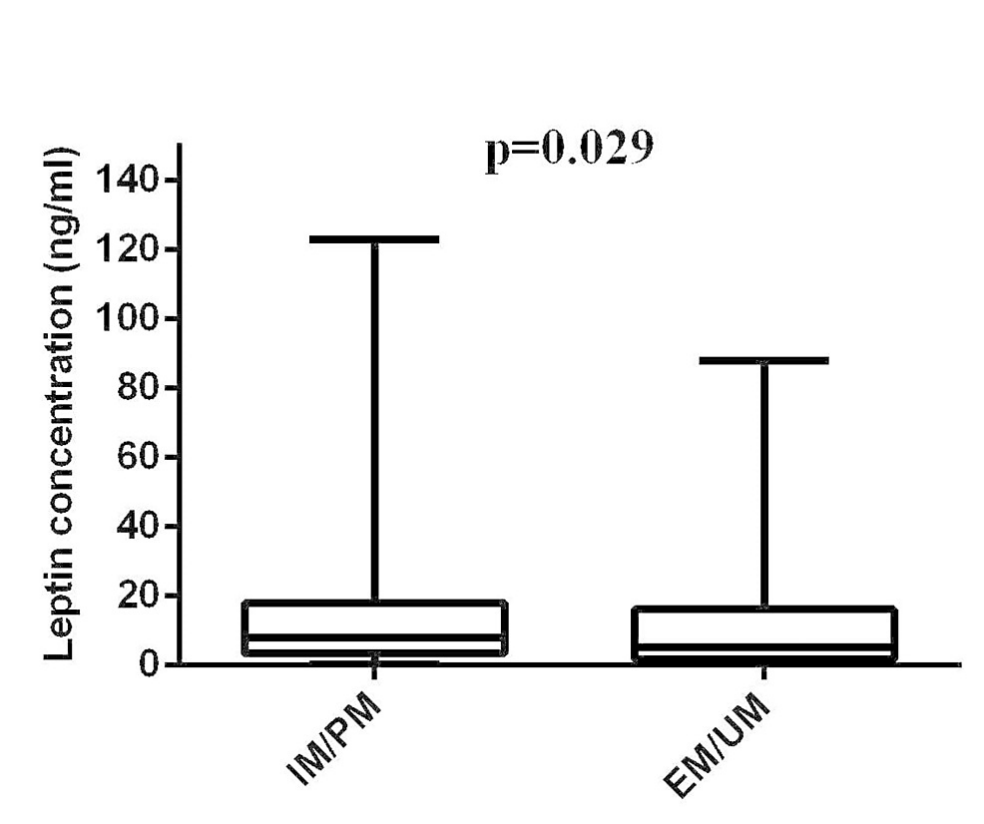
Comparison of leptin levels (ng/ml) between IM/PM (n = 118) and EM/UM groups (n = 86) IM: intermediate metabolizer; PM: poor metabolizer; EM: extensive metabolizer; UM: ultra metabolizer.

The proportion (%) of IM/PM was 57.8 (118). After comparing adipocytokines levels between IM/PM and EM/UM groups, leptin levels were found to be increased in IM/PM (median = 8 ng/ml; IQR = 3.4-18.0) with respect to EM/UM (median = 5.3 ng/ml; IQR = 1.7-16.2; p = 0.029) (Figure 3).

Effect of Genetic Variants and Adipocytokines (Leptin and Total Adiponectin) Levels on Clinical Outcome

**Figure 4:**
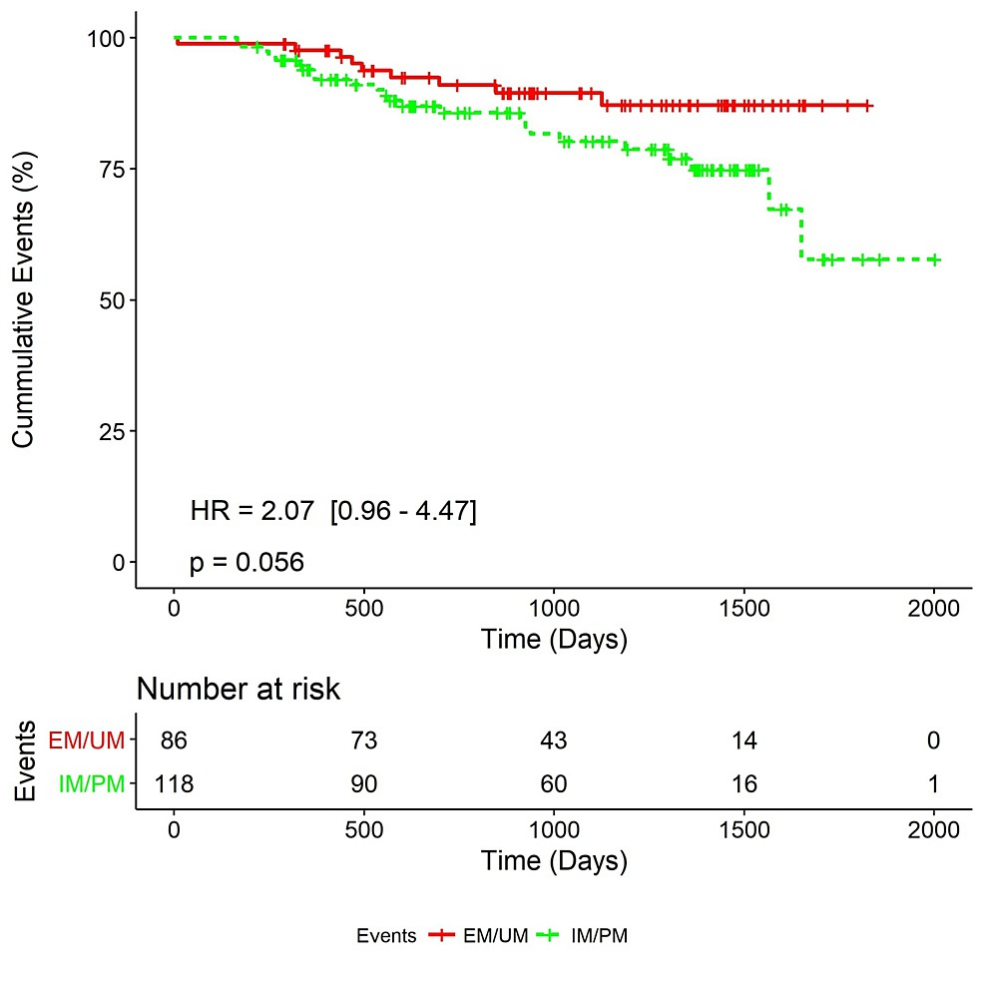
Cumulative Kaplan-Meier survival curve showing the impact of CYP2C (IM/PM) metabolizers on the composite vascular event (stroke/TIA/myocardial infarction/death) (n = 204) IM: intermediate metabolizer; PM: poor metabolizer; EM: extensive metabolizer; UM: ultra metabolizer; TIA: transient ischemic attack.

A follow-up was carried out with 174 (85.2%) patients who were queried for any recurrence of ischemic stroke, TIAs, and death due to vascular causes. Patients unable to visit the hospital were followed up telephonically. The endpoint was TIA/stroke/any vascular death. A total of 33 (19%) patients reached the composite endpoint of death, recurrent TIAs, and ischemic events. The median follow-up time was 34 months (IQR: 19-46). The percentage of composite outcome (TIA/stroke/any vascular death) was higher in IM/PM (72.7%) than in EM/UM (27.3%). A trend of association was reported between the occurrence of composite vascular events and IM/PM phenotypes (24 vs. nine events, HR = 2.07 (95% CI = 0.96-4.47), p = 0.056) (Figure 4).

## Discussion

In our study, we have evaluated the effect of polymorphisms of CYP2C19 (allele *2 (rs424485), allele *3 (rs4986893), allele *4 (rs28399504), and allele *17 (rs12248560)) on the occurrence of stroke, occurrence of the composite endpoint (TIA/ischemic stroke/vascular death), death vs. live, and recurrence of stroke/TIA. From the knowledge of the literature, these genotypes were categorized into poor/intermediate metabolizers and extensive/ultra metabolizers [12]. Adiponectin and leptin levels were evaluated among patients with EM/UM and PM/IM genotypes.

Stroke vs. no stroke (control)

In our study, no significant association was observed between the occurrence of stroke and SNP1. SNP2 showed a significant positive association with the occurrence of stroke in both of these two models (recessive and the log additive model). No significant covariate effect was seen with sex when adjusted by age. The AC (OR = 1.75 (1.08-2.83), p = 0.024) and GT haplotypes (OR = 3.33 (1.53-7.22), p = 0.0026) were found to be strongly associated with the occurrence of stroke (global haplotype association p-value: 0.0062). Among females, the haplotype phenotype interaction was not observed; however, haplotype phenotype interaction was evident among males, and compared to the GC haplotype, AC and GT haplotypes were significantly associated with the occurrence of stroke (after adjusting for age). In previous literature also, Cyp2C19 polymorphism (G681A) was found to be associated with both occurrence and recurrence of cerebral ischemic stroke [13]. Another study found an association between CYP1A1 activity and the occurrence of stroke [14]. Findings from these studies support our findings that CYP450 polymorphisms can be directly associated with stroke. CYP450 enzymes are involved in the metabolism of many endogenous molecules, which may explain the findings.

Stroke patients: composite outcome

Among stroke patients, while evaluating the association between SNP1 and the occurrence of the composite outcome, the best-performing models in terms of lowest AIC and BIC score were the dominant model (2.31 (0.99-5.35), p = 0.043) and log-additive model (1.82 (1.02-3.27), p = 0.042). Interestingly, both these models showed a positive association between SNP1 and the occurrence of composite outcomes. However, no association was seen between SNP2 and the occurrence of composite outcomes. While evaluating the different haplotypes of SNP1 and SNP2 and their association with the occurrence of the composite outcome, the AC haplotype was significantly associated with the occurrence of the primary outcome (OR = 2.27 (1.17-4.41), p = 0.016, global haplotype association p-value: 0.044). While evaluating the interaction between haplotypes, composite outcome, and impact of important covariates (sex), no significant covariate effect was observed after adjusting for age.

All stroke patients: alive vs. dead

A significant positive association was seen between the occurrence of the final outcome (death) and SNP1 (OR = 2.35 (1.13-4.90), p = 0.021). However, no association was seen between the occurrence of the final outcome (death) and SNP2 (OR = 0.53 (0.20-1.45), p = 0.19).

Among different haplotypes, the AC haplotype was found to be associated with death (OR 2.73 (1.20-6.22), p = 0.018, global haplotype association p-value: 0.039). No significant interaction was seen between the occurrence of the final outcome, haplotype, and sex (after adjustment by age).

All live stroke patients: recurrence vs. no recurrence

No association was seen with recurrence and SNP1 (OR = 0.38 (0.10-1.40), 0.12) and SNP2 (OR = 0.83 (0.44-1.59), p = 0.58). Among live stroke patients, no significant association was seen between any of the haplotypes and recurrence. No significant covariate effect of age was observed after adjustment for age.

Adiponectin and leptin levels and occurrence of stroke and metabolic phenotype

Significant higher levels of leptin (ng/ml) and lower levels of total adiponectin (µg/ml) were observed between patients and controls. The levels of leptin and adiponectin levels were higher in females compared to males. After comparing adipocytokines levels between IM/PM and EM/UM groups, leptin levels were found to be increased in IM/PM with respect to EM/UM. The percentage of composite outcome (TIA/stroke/any vascular death) was higher in IM/PM (72.7%) than in EM/UM (27.3%). A trend of association was seen between the occurrence of composite vascular events and IM/PM phenotypes (24 vs. nine events, HR = 2.07 (95% CI = 0.96-4.47), p = 0.056). A subsequent study with a higher sample will be helpful in further delineating the effect size.

Up to now, several studies showed the role of CYP2C enzymes on the metabolism of xenobiotic agents and drugs. However, to the best of our knowledge, an association between the CYP2C genetic system and dysregulation of adipocytokine in relation to ischemic stroke has not been studied earlier. Among all devastating neurological diseases, stroke is the leading one, which causes death or disability in developing countries, including India [15]. Stroke pathology is complex and various cytokines are formed when the cascade of two mechanisms, namely, inflammation and atherosclerosis, is in progress [6,16,17]. Leptin and adiponectin are adipose-derived cytokines that have manifold effects on human biology. Functionally, leptin is pleiotropic in nature; it shows pro-atherogenic effects and promotes thrombosis both in vivo and in vitro conditions through the presence of its receptor on platelet surfaces [18]. Adiponectin exhibited anti-atherogenic and anti-thrombotic properties [19]. Dysregulation of these two adipocytokines is associated with all types of strokes and the same has been reported in the current study while comparing the adipocytokines levels between ischemic attack patients and healthy controls. CYP genes are highly polymorphic and previous literature suggests that increased serum (or plasma) levels of pro-inflammatory cytokines (i.e., IL-1, IL-6, and tumor necrosis factor-α) are influenced by the expression of the CYP system [20,21]. A study by Akasaka et al. proved that CYP2C19 variants affect microvascular dysfunction and CYP2C19 poor metabolizers secrete the increased levels of high-sensitivity C-reactive protein (hs-CRP) that further suggests CYP system involvement in inflammation [22]. In the current study, the prevalence of CYP2C19 IM/PM phenotypes is reported at a frequency of 57.8%, which is higher than previous reports from Asia [12]. In previous reports, CYP2C19*2 is also determined as a risk factor gene associated with recurrent vascular events in cardiovascular and cerebrovascular diseases; however, the underlying mechanism is unclear [13,23]. The current study also found CYP2C19 as a candidate gene that increased the risk of vascular events in ischemic stroke patients.

The current study had a few limitations. Firstly, a single-time assessment of leptin and adiponectin levels was done, which is insufficient to understand changes in the dynamics of inflammation and thrombosis over the follow-up period. Moreover, due to inadequate sample size, the involvement of the CYP enzyme system in adipocytokines production is inconclusive.

## Conclusions

With regard to the occurrence of stroke, SNP2 showed a significant positive association. Haplotypes (allele *2/*17, i.e., SNP1/SNP2) AC (OR = 1.75 (1.08-2.83), p = 0.024) and GT (OR = 3.33 (1.53-7.22), p = 0.0026) were strongly associated with the occurrence of stroke even after adjustment for age and sex (global haplotype association p-value: 0.0062). Haplotype phenotype gender interaction was evident. Among stroke patients, with regard to composite outcome, only SNP1 showed a positive association. The AC haplotype was significantly associated with the occurrence of composite outcome (OR = 2.27 (1.17-4.41), p = 0.016). Among stroke patients, a significant positive association was seen between death and SNP1 (OR = 2.35 (1.13-4.90), p = 0.021) and AC haplotype (OR = 2.73 (1.20-6.22), p = 0.018). However, none of the SNPs or haplotypes showed any association with recurrence. Significantly higher leptin and lower adiponectin levels were observed among stroke patients compared to controls. Leptin levels were found to be increased in IM/PM phenotypes. IM/PM phenotypes showed a higher incidence of occurrence of composite vascular events.

To conclude, CYP2C19 polymorphisms may play a significant role in the pathogenesis of stroke. Leptin could serve as a prominent biomarker of atherosclerosis and inflammation in the early post-stroke period; however, further study is warranted with a larger sample size. Upregulation and downregulation of leptin and adiponectin in ischemic stroke could suggest their importance as future pharmacological targets of atherosclerosis and inflammation; however, this dysregulation due to enzymatic action of CYP2C19 phenotypes needs to be explored further in a large prospective study on ischemic stroke.

## Appendices

Supplementary tables

**Table 21:** Supplementary tables A1 to A.9 PCR: polymerase chain reaction; SNP: single nucleotide polymorphism; HWE: Hardy-Weinberg equilibrium; AIC: Akaike information criterion; BIC: Bayes information criterion.

Supplementary Table A1: TaqMan genotyping assays																																									
Gene/reference SNP ID (rs)									#Context sequence (VIC/FAM) allele 1/allele 2																																
CYP2C19*2 rs 4244285									TTCCCACTATCATTGATTATTTCCC[A/G]GGAACCCATAACAAATTACTTAAAA MAF/Wild																																
CYP2C19*3 rs 4986893									ACATCAGGATTGTAAGCACCCCCTG[A/G]ATCCAGGTAAGGCCAAGTTTTTTGC MAF/Wild																																
CYP2C19*4 rs 28399504									GTCTTAACAAGAGGAGAAGGCTTCA[A/G]TGGATCCTTTTGTGGTCCTTGTGCT Wild/MAF																																
CYP2C19*17 rs12248560									AAATTTGTGTCTTCTGTTCTCAAAG[C/T]ATCTCTGATGTAAGAGATAA Wild/MAF																																
*MAF means minor allele frequency; # context sequences obtained from www.thermofisher.com																																									
Supplementary Table A2: PCR reaction mixture details																																									
PCR component								Volume per reaction^#^ (10 μl)																		Final concentration															
2X TaqMan Universal Master mix								5																		1X															
20X or 40X genotyping assay (primer and probe)								0.5 or 0.25																		1X															
Genomic DNA								2																		≥5-25 ng															
Autoclaved milli-Q water								2.5 or 2.75																		-															
^#^MicroAmp Fast 96 well reaction plate (0.1 ml) (Applied Biosystems, Waltham, MA).																																									
Supplementary Table A3: All stroke patients: alive vs. dead. Haplotype and interaction with sex (adjusted by age)																																									
											Haplotype within sex (n = 204)																	Sex within haplotype (n = 204)													
Haplotype			Frequency								Female								Male									Female							Male						
											OR (95% CI)								OR (95% CI)									OR (95% CI)							OR (95% CI)						
GC			0.4673								1.00								1.00									1.00							1.38 (0.12-15.56)						
AC			0.317								4.93 (0.98-24.73)								2.14 (0.82-5.63)									1.00							0.60 (0.17-2.10)						
AT			0.0286								0.00 (-Inf-Inf)								0.00 (-Inf-Inf)									1.00							Inf						
GT			0.1871								0.95 (0.10-9.08)								0.98 (0.27-3.47)									1.00							1.42 (0.13-15.93)						
Supplementary Table A4: Allelic frequency, genotype frequency, and Hardy-Weinberg equilibrium: All alive patients: No recurrence vs. recurrence																																									
SNP		Allele/genotype														All subjects (n = 185)									No recurrence									Recurrence							
																Count		Proportion							Count						Proportion			Count						Proportion	
SNP1		Allele								G						248		0.67							165						0.67			83						0.68	
										A						122		0.33							83						0.33			39						0.32	
		Genotype								A/A						17		0.09							14						0.11			3						0.05	
										G/A						88		0.48							55						0.44			33						0.54	
										G/G						80		0.43							55						0.44			25						0.41	
		HWE														0.40									1.00									0.081							
SNP2		Allele								C						287		0.78							192						0.77			95						0.78	
										T						83		0.22							56						0.23			27						0.22	
		Genotype								C/C						109		0.59							72						0.58			37						0.61	
										C/T						69		0.37							48						0.39			21						0.34	
										T/T						7		0.04							4						0.03			3						0.05	
		HWE														0.4									0.31									1.00							
Supplementary Table A5: All alive patients: No recurrence vs. recurrence: Genetic association data (n = 185, adjusted by sex and age)																																									
SNP	Model							Genotype						No recurrence							Recurrence						OR						P-value						AIC		BIC
SNP1	Co-dominant							G/G						55 (44.4%)							25 (41%)						1.00						0.19						239.5		255.6
								A/G						55 (44.4%)							33 (54.1%)						1.35 (0.71-2.57)														
								A/A						14 (11.3%)							3 (4.9%)						0.45 (0.12-1.72)														
	Dominant							GG						55 (44.4%)							25 (41%)						1.00						0.64						240.6		253.5
								A/G-A/A						69 (55.6%)							36 (59%)						1.16 (0.62-2.17)														
	Recessive							G/G-A/G						110 (88.7%)							58 (95.1%)						1.00						0.12						238.4		251.2
								A/A						14 (11.3%)							3 (4.9%)						0.38 (0.10-1.40)														
	Over dominant							G/G-A/A						69 (55.6%)							28 (45.9%)						1.00						0.18						239.1		252
								A/G						55 (44.4%)							33 (54.1%)						1.52 (0.82-2.83)														
	Log additive							--																			0.92 (0.57-1.50)						0.75						240.7		253.6
SNP2	Co-dominant							C/C						72 (58.1%)							37 (60.7%)						1.00						0.81						242.4		258.5
								C/T						48 (38.7%)							21 (34.4%)						0.85 (0.44-1.64)														
								T/T						4 (3.2%)							3 (4.9%)						1.34 (0.28-6.38)														
	Dominant							C/C						72 (58.1%)							37 (60.7%)						1.00						0.72						240.7		253.6
								C/T-T/T						52 (41.9%)							24 (39.3%)						0.89 (0.47-1.67)														
	Recessive							C/C-C/T						120 (96.8%)							58 (95.1%)						1.00						0.66						240.7		253.5
								T/T						4 (3.2%)							3 (4.9%)						1.42 (0.30-6.66)														
	Over dominant							C/C-T/T						76 (61.3%)							40 (65.6%)						1.00						0.58						240.5		253.4
								C/T						48 (38.7%)							21 (34.4%)						0.83 (0.44-1.59)														
	Log additive																										0.96 (0.56-1.65)						0.87						240.8		253.7
Supplementary Table A6: All alive stroke patients: No recurrence vs. recurrence: Sex and SNP covariate interaction (sex within SNP)																																									
						Sex within SNP (n = 185, adjusted by age)																																			
SNP1																						Non-recurrent							Recurrent							OR					
						G/G							Female									13							7							1.00					
													Male									42							18							0.78 (0.26-2.28)					
						A/G							Female									9							9							1.00					
													Male									46							24							0.51 (0.18-1.47)					
						A/A							Female									3							1							1.00					
													Male									11							2							0.52 (0.03-7.95)					
						Test for interaction in the trend = 0.67																																			
SNP2						C/C							Female									16							10							1.00					
													Male									56							27							0.75 (0.30-1.89)					
						C/T							Female									7							6							1.00					
													Male									41							15							0.42 (0.12-1.47)					
						T/T							Female									2							1							1.00					
													Male									2							2							2.00 (0.09-44.37)					
						Test for interaction in the trend = 0.81																																			
Supplementary Table A7: All alive stroke patients: No recurrence vs. recurrence: Sex and SNP covariate interaction (SNP within sex)																																									
SNP					Gender										Genotype								Non-recurrent							Recurrent							OR				
SNP1					Female										G/G								13							7							1.00				
															A/G								9							9							1.84 (0.50-6.78)				
															A/A								3							1							0.59 (0.05-6.85)				
					Male										G/G								42							18							1.00				
															A/G								46							24							1.22 (0.58-2.56)				
															A/A								11							2							0.40 (0.08-2.01)				
					Test for interaction in the trend = 0.86																																				
SNP2					Female										C/C								16							10							1.00				
															C/T								7							6							1.33 (0.35-1.58)				
															T/T								2							1							0.78 (0.06-9.77)				
					Male										C/C								56							27							1.00				
															C/T								41							15							0.75 (0.35-1.58)				
															T/T								2							2							2.07 (0.28-15.48)				
					Test for interaction in the trend = 0.58																																				
Supplementary Table A8: All alive stroke patients: No recurrence vs. recurrence: Haplotype association																																									
S. no.							SNP1										SNP2							Frequency								OR						P-value			
1							G										C							0.479								1.00						--			
2							A										C							0.2967								1.09 (0.61-1.93)						0.78			
3							G										T							0.1913								1.12 (0.60-2.11)						0.72			
4							A										T							0.033								0.32 (0.04-2.67)						0.29			
Global haplotype association p-value: 0.68																																									
Supplementary Table A9: Haplotype and interaction with sex (adjusted by age)																																									
												Haplotype within sex (n = 204)																Sex within haplotype (n = 204)													
Haplotype				Frequency								Female								Male								Female							Male						
												OR (95% CI)								OR (95% CI)								OR (95% CI)							OR (95% CI)						
GC				0.4789								1.00								1.00								1.00							0.68 (0.18-2.48)						
AC				0.2967								1.08 (0.32-3.58)								1.09 (0.57-2.06)								1.00							0.68 (0.25-1.85)						
AT				0.033								1.32 (0.08-21.63)								0.00 (-Inf-Inf)								1.00							0.00 (-Inf-Inf)						
GT				0.1913								1.05 (0.34-3.22)								1.20 (0.57-2.52)								1.00							0.77 (0.25-2.39)						
